# Practices among General Practitioners in Rheumatoid Arthritis (GEPRA-I): results of a region-wide online survey

**DOI:** 10.1186/s12875-022-01744-5

**Published:** 2022-06-03

**Authors:** Anne-Laure Yailian, Charline Estublier, Aurélie Fontana, Emmanuelle Vignot, Cyrille Confavreux, Roland Chapurlat, Humbert de Fréminville, Audrey Janoly-Dumenil

**Affiliations:** 1 Service de Pharmacie, Hôpital Edouard Herriot, Hospices Civils de Lyon, 5 place d’Arsonval, 69003 Lyon, France; 2 EA 4129, Parcours Santé Systémique P2S, Université Claude Bernard Lyon 1, 7-11 rue Guilllaume Paradin, 69008 Lyon, France; 3 Service de Rhumatologie, Hospices Civils de Lyon, Lyon, France; 4 INSERM UMR 1033, Faculté de Médecine Laennec, Université Claude Bernard Lyon 1, 7-11 rue Guillaume Paradin, 69008 Lyon, France; 5 Service de Rhumatologie, Hôpital Edouard Herriot, Hospices Civils de Lyon, 5 place d’Arsonval, 69003 Lyon, France; 6 Faculté de Pharmacie, Université Claude Bernard Lyon 1, 8 avenue Rockefeller, 69008 Lyon, France

**Keywords:** Rheumatoid arthritis, General practitioners, Multidisciplinary, Survey

## Abstract

**Background:**

To assess current practice regarding the management of rheumatoid arthritis patients among general practitioners of a French region, and their perception about the deployment of a multidisciplinary collaboration.

**Methods:**

A cross-sectional online survey was sent to the general practitioners of a French region. The questionnaire comprised of 3 sections to collect data regarding 1/demographics, 2/practice and knowledge in rheumatoid arthritis, and 3/perception about the deployment of a multidisciplinary collaboration.

**Results:**

1/A total of 247 general practitioners (M/F ratio: 1.4; mean age: 46.7 years) completed the survey. 2/More than half of general practitioners believed that their role was very or extremely important in disease diagnosis (72.5%), and management of comorbidities (67.2%). Among respondents, 6.1% considered that they did not face any difficulty concerning the patient management and 61.5% had already identified causes of non-adherence. 3/A total of 151 (61.1%) general practitioners were willing to participate in a multidisciplinary programme to improve medication adherence in rheumatoid arthritis.

**Conclusions:**

General practitioners are motivated to contribute to an overall management of rheumatoid arthritis patients. Nevertheless, they need professional education about rheumatoid arthritis treatment and training in motivational interviews before getting involved in a multidisciplinary collaboration.

**Supplementary Information:**

The online version contains supplementary material available at 10.1186/s12875-022-01744-5.

## Introduction

Rheumatoid arthritis (RA) is one of the most common chronic and inflammatory disease in rheumatology and affects 0.5% to 1% of the worldwide population [1]. In France, its prevalence is estimated at 0.35% [2]. Therapeutic strategies for RA management have significantly improved over the past decades [3]. In 2019, the French Society for Rheumatology (*Société Française de Rhumatologie*, SFR) has updated its recommendations in order to provide optimal care for RA patients [4], based on EULAR guidelines (European Alliance of Associations for Rheumatology) [5]. These recommendations address the diagnosis, treatment, follow-up, management of remissions, and management of comorbidities related to RA. Notably, the care of RA patients should rely on a rheumatologist supported by a multidisciplinary team [4, 6]. Establishing diagnosis and initiating treatment as early as possible is emphasised in the SFR recommendations, highlighting the crucial role of the general practitioner (GP) in the early detection of RA. For this purpose, GPs should be trained in recognising the signs suggestive of early inflammatory joint disease, and rheumatologists should be available to assess rapidly the referred patients [7]. Also, rheumatologists and GPs should work together to provide a personalised and coordinated follow-up [4, 8]. Usually, GPs are also involved in monitoring treatments and managing comorbidities; in coordination with rheumatologists, they may also be in charge of renewing and adjusting medications. To our knowledge, no recent study has focused on the practices of GPs regarding RA patient care in France. Thus, the objectives of the present study were to determine the knowledge and practices of GPs in RA and investigate their perceptions and motivation to develop a multidisciplinary collaboration.

## Methods

### Ethics approval

Ethics approval was obtained from the ethics committee of the *Université Claude Bernard Lyon 1* (No. IRB: 2019–05-21–03) on the 21th May 2019.

### Study design

This study was a cross-sectional online survey, and was part of the GEPRA project (General Practitioners in Rheumatoid Arthritis) that aimed to analyse the practices of GPs in RA and explore their barriers and drivers to be involved in a multidisciplinary patient support programme. The whole project has been designed to help in the future development of a multidisciplinary intervention to enhance medication adherence in RA.

### Survey

A 35-question online survey (Additional files 1 and 2) was developed to address the stated aims of the investigation, by rheumatologists, general practitioners and pharmacists. The questionnaire was designed following recommendation from the Checklist for Reporting Results of Internet E-Surveys (CHERRIES) [9] (Additional file 3), and included a combination of multiple-choice questions, close- and open-ended questions, and 5-point Likert scale questions [10]. The questions were arranged into 3 distinct sections. The first section consisted of 8 questions regarding the demographic information and practice characteristics of participants (gender, age, practice location, and years of work experience). The second section contained 20 questions assessing the GPs’ practice and knowledge in RA. Participants were specifically asked to estimate their knowledge of RA therapeutic strategies on a scale ranging from 0 to 10 (a high score was indicative of sufficient knowledge). The third section related to community-hospital and multidisciplinary collaboration was composed of 7 questions. The questions were uploaded onto Claroline Connect (*Université Claude Bernard Lyon 1*, Lyon, France) for distribution and data collection. Four GPs and four rheumatologists pretested the questionnaire for its feasibility and understandability. No major adjustment was necessary.

### Participants

GPs were eligible to participate in the study if they were current practicing GPs in the Auvergne-Rhône-Alpes (AurRA) region. The survey was sent by the URPS (*Union Régionale des Professionnels de Santé)* on the 10^th^ July 2019 by electronic mail with a cover letter stating the purpose of the study to GPs in the AuRA region. Participation in the study was voluntary and indicative of consent. Respondents had the option to opt out at any time. All responses were recorded anonymously and no identifying information was collected.

The survey was open between the 10^th^ July 2019 and the 10^th^ October 2019, and took approximately 10 min to complete. All answers were required to finish the questionnaire, except for 2 open-ended questions that were adaptive (some questions were displayed only conditionally based on the answers to the previous question). Participants could use a back function to change a response if necessary. The answers from returned questionnaires were directly entered into an online database. One mail reminder with the survey link was sent to all potential participants in September 2019.

Upon survey completion, each participant was invited to download the 2019 SFR recommendations on the management of RA patients and a leaflet on the main characteristics of Disease Modifying Anti-Rheumatic Drugs (DMARDs), and was given the option to contact the research team in case of additional questions or comments. Respondents were not compensated for their participation.

### Data analysis

For close-ended and 5-point Likert scale questions, a descriptive data analysis was performed using SPSS® Statistics software (Version 21, IBM® Corporation, Armonk, NY, USA) and data were expressed as mean or count (percentage).

Possible duplicate responses were identified according to the responses to the socio-demographic aspects. A participation rate was defined as the ratio of [the number of GPs who had started the questionnaire]/[the number of GPs who received the email sent by URPS]. Only complete responses from participants were analysed. A completion rate was defined as the ratio of [the number of GPs who finished the survey]/[the number of GPs who started the questionnaire].

Relevant variables selected by the authors were tested by univariate analysis (Chi-square test for categorical variables, and Student-test for continuous variables) to detect a possible correlation with GP motivation to participate in a multidisciplinary programme. GP motivation was estimated from the answer to the binary question ‘Would you be willing to participate in a multidisciplinary programme in collaboration with hospital professionals and community pharmacists to improve the medication adherence of your patients?’. Variables significantly correlated with GP motivation (*P* < 0.2) in univariate analysis were included in a multivariable model. The multivariate analysis was performed by logistic regression. Results were expressed as odds ratios (OR) and 95% confidence intervals (CI). A value of *P* < 0.05 was considered statistically significant.

For open-ended questions, answers were coded according to their common themes (ALY), reviewed by a second author (AJD), and then summarised.

## Results

Following the exclusion of invalid addresses, the survey was successfully distributed to 4,644 GPs in the AuRA region and 383 (8.2%) of them responded. The survey completion rate was 64.5% (247/383), and there was no duplicate response. Women were predominant among responders (144/247, 58.3%). The mean age of the participants was 46.7 years (Table 1).

**Table 1:** Demographic characteristics of the GPs participating in the survey

	**n**	**(%)**
**Gender**		
Male	103	41.7
Female	144	58.3
**Age group (years)**		
[25; 29]	5	2.0
[30; 34]	40	16.2
[35; 39]	45	18.2
[40; 44]	31	12.6
[45; 49]	25	10.1
[50; 54]	14	5.7
[55; 59]	37	15.0
[60; 64]	42	17.0
[65; 69]	8	3.2
**Seniority (years in general practice)**		
< 5	54	21.9
[5; 10]	55	22.3
[10; 20]	50	20.2
[20; 30]	41	16.6
> 30	47	19.0
**Practice structure**		
Medical practice alone	54	21.9
Medical group practice	135	54.7
Multidisciplinary health centre	54	21.9
Employee in a health care institution	2	0.8
Other	2	0.8
**Numbers of patients per week registered at the GP practice**		
< 50	13	5.3
[50; 100]	126	51.0
[101; 200]	98	39.7
> 200	10	4.0
**Practice location**		
Urban area	84	34.0
Semi-rural area	105	42.5
Rural area	58	23.5
**Practice department**		
Ain	30	12.1
Allier	13	5.3
Ardèche	17	6.9
Cantal	3	1.2
Drôme	24	9.7
Isère	34	13.8
Loire	16	6.5
Haute-Loire	9	3.6
Puy de Dôme	18	7.3
Rhône	43	17.4
Savoie	20	8.1
Haute-Savoie	20	8.1

Abbreviations: *GP* general practitioner

### Practices and knowledge in rheumatoid arthritis

**Fig. 1:**
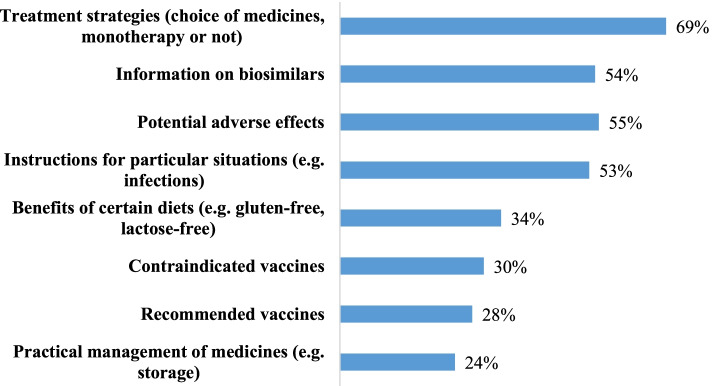
Main difficulties of GPs in the therapeutic management of RA patients (*N* = 247). Abbreviations: GP, general practitioner, RA, rheumatoid arthritis

Regarding the follow-up of patients, 152 (61.5%) responding GPs reviewed less than 5 RA patients/year. Their consultations with them were regular (every 3 to 6 months) for 179 (72.5%) GPs. According to responders, specialised rheumatology care for their RA patients was carried out: mostly in the hospital (*n* = 88, 35.6%), mostly in the community (*n* = 77, 31.2%), in the community and in the hospital (n = 82, 33.2%). Among the participants, 62 (25.1%) had already encountered difficulties in obtaining specialised rheumatology opinions. Regarding their role, GPs believed that it was very or extremely important particularly in disease diagnosis (*n* = 179, 72.5%), management of comorbidities (*n* = 166, 67.2%), supporting patients in the treatment management (*n* = 115, 46.6%), and treatment tolerance monitoring (*n* = 109, 44.1%; Table 2). Overall, 180 (72.9%) responders reported that they evaluated the medication adherence of patients during consultations and 152 (61.5%) had already identified causes of non-adherence. Among the 228 causes identified, the main ones were the adverse effects suffered (*n* = 106, 46.5%), beliefs about the drug (*n* = 52, 22.8%), the constraints associated with its use (*n* = 19; 8.3%), the duration of treatment (*n* = 11, 4.8%), the lack of information (*n* = 9, 3.9%), administration modalities (*n* = 8, 3.5%), and ineffectiveness (*n* = 7, 3.1%). Regarding the RA patient management, 15 (6.1%) GPs considered that they did not face any particular difficulty. The majority of respondents (*n* = 227, 91.9%) was not aware of the updating of the RA recommendations. Their main difficulties were mainly related to treatment strategies (*n* = 170, 68.8%), information on biosimilars (*n* = 133, 53.8%), potential adverse effect (*n* = 135, 54.7%), and instructions for particular situations (*n* = 130, 52.6%; Fig. 1). GPs self-assessed their knowledge about the current treatment strategy for RA at 4.7/10 on average. A majority of GPs (*n* = 224, 90.7%) reported that they were uncertain about new synthetic targeted therapies and 156 (63.2%) declared that they were uncertain about biological targeted therapies. For 143 (57.9%) GPs, targeted therapies (biological or synthetic) were to be preferably used in monotherapy. Few GPs (*n* = 32, 13.0%) had already witnessed adverse events (AEs) in patients under targeted therapy. Among the 42 AEs reported, the most frequent ones were infections (*n* = 18, 42.9%), asthenia (*n* = 7, 16.7%), injection site reaction/hypersensitivity (*n* = 6, 14.3%), and digestive disorders (*n* = 5, 11.9%). According to GPs, patients' knowledge was insufficient or very insufficient regarding the mechanisms of action (*n* = 171, 69.2%), adverse effects (*n* = 136, 55.1%), vaccinations (*n* = 159, 64.4%), and biosimilars (*n* = 170, 68.9%; Table 3). Finally, 170 (68.8%) GPs declared they regularly discussed lifestyle recommendations (including physical activity, smoking cessation, and healthy diet) with their patients. The main comorbidities mentioned by GPs and their risk factors were the vaccination status (*n* = 206, 83.4%), cardiovascular risk factors (*n* = 187, 75.7%), cancer screening tests (*n* = 164, 66.4%), and the risk of osteoporosis (*n* = 153, 61.9%; Table 4).

**Table 2:** Perception of GPs regarding their role in the care of RA patients

	**n** **(%)**				
	**Not at all important**	**Unimportant**	**Relatively important**	**Very important**	**Extremely important**
**Disease diagnosis**	1 (0.4%)	7 (2.8%)	60 (24.3%)	136 (55.1%)	43 (17.4%)
**Disease monitoring**	3 (1.2%)	67 (27.1%)	113 (45.7%)	57 (23.1%)	7 (2.8%)
**Treatment effectiveness monitoring**	5 (2.0%)	75 (30.4%)	105 (42.5%)	54 (21.9%)	8 (3.2%)
**Treatment tolerance monitoring**	2 (0.8%)	18 (7.3%)	118 (47.8%)	87 (35.2%)	22 (8.9%)
**Prescription refills of DMARDs**	23 (9.3%)	84 (34.0%)	86 (34.8%)	45 (18.2%)	9 (3.6%)
**Therapeutic adjustments (corticosteroids or DMARDs)**	24 (9.7%)	111 (44.9%)	78 (31.6%)	32 (13.0%)	2 (0.8%)
**Management of co-morbidities**	1 (0.4%)	11 (4.5%)	69 (27.9%)	122 (49.4%)	44 (17.8%)
**Supporting patients in the treatment management**	2 (0.8%)	38 (15.4%)	92 (37.2%)	93 (37.7%)	22 (8.2%)

Abbreviations: *GP* general practitioner, *RA* rheumatoid arthritis

**Table 3:** Patients’level of knowledge regarding DMARDs according to general practitioners

	**n** **(%)**				
	**Very insufficient**	**Insufficient**	**No opinion**	**Appropriate**	**Excellent**
**Mechanism of action**	67 (27.1%)	104 (42.1%)	61 (24.7%)	13 (5.3%)	2 (0.8%)
**Potential adverse effects and associated behaviours**	15 (6.1%)	121 (49.0%)	44 (17.8%)	66 (26.7%)	1 (0.4%)
**Frequency of administration**	9 (3.6%)	36 (14.6%)	35 (14.2%)	157 (63.6%)	10 (4.0%)
**Vaccination status**	31 (12.6%)	128 (51.8%)	32 (13.0%)	50 (20.2%)	6 (2.4%)
**Biosimilars**	72 (29.1%)	98 (39.7%)	66 (26.7%)	10 (4.0%)	1 (0.4%)
**Self-injection**	25 (10.1%)	64 (25.9%)	83 (33.6%)	72 (29.1%)	3 (1.2%)

**Table 4:** Main comorbidities and risks factors mentioned by general practitioners

	**n**	**(%)**
Vaccination status	206	83.4%
Cardiovascular risk factors	187	75.7%
Cancer screening tests	164	66.4%
Risk of osteoporosis	153	61.9%
Depression	101	40.9%
Oral health	94	38.1%
Pulmonary damage	92	37.2%
Digestive disorder	65	26.3%
None of the items above	11	4.5%

### Community-hospital and interprofessional collaboration

The follow-up of RA patients was reported as sufficiently coordinated between health professionals by less than half of the GPs (100, 40.5%). Among the respondents, 106 (42.9%) considered that they did not receive enough information from the rheumatologist regarding follow-up and modifications and monitoring of background treatment. There were 151 (61.1%) responders reporting to be willing to participate in a multi-professional programme with hospital professionals and the community pharmacist to improve patient medication adherence. The barriers identified to their participation were: lack of time (*n* = 84, 42.0%), lack of motivation (*n* = 25, 12.5%), lack of training (*n* = 19, 9.5%), lack of added value (*n* = 4, 2.0%), small number of patients concerned among the patient population (*n* = 54, 27.0%), and lack of willingness of patients (*n* = 14, 7.0%). The majority of GPs (*n* = 236, 95.5%) found it useful to have a secure electronic messaging system to exchange with other professionals about their patients. There were 104 (42.1%) GPs reporting interest in a training in the practice of motivational interviewing to improve medication adherence. Also, 166 (67.2%) GPs were willing to dedicate additional time during a consultation with a patient to explore and enhance medication adherence, with appropriate training and remuneration.

### Factors influencing motivation of GPs to get involved in a multidisciplinary programme

The univariate analysis identified 7 variables that were associated with GP motivation. Among these variables, 3 were significant in the multivariate analysis: causes of medication non-adherence already identified (OR = 1.95, 95% CI [1.03; 3.69], *p* = 0.04), interest in training in the practice of motivational interviewing to improve medication adherence (OR = 3.83, 95% CI [1.91; 7.69], *p* < 0.01), and interest in providing additional time to explore and improve medication adherence with financial compensation (OR = 3.11, 95% CI [1.58; 6.13], *p* < 0.01; Table 5).

**Table 5:** Association of different variables with the GP motivation to participate in a multidisciplinary program: results of the univariate and multivariate analyses

	**Univariate model**		**Multivariate model**		
**Variable**	**χ**^**2**^** or *t***^***a***^	***P-value***	**OR**	**[95% CI]**	***P-value***
**Gender**	3.60	0.04			
Male			1.00	-	-
Female			1.06	[0.55; 2.05]	0.86
**Age**	16.49	0.04			
[25; 29]			1.00	-	-
[30; 34]			2.31	[0.25; 21.77	0.46
[35; 39]			0.93	[0.11; 8.20]	0.95
[40; 44]			0.51	[0.06; 4.76]	0.56
[45; 49]			1.00	[0.10; 9.75]	1.00
[50; 54]			0.82	[0.08; 8.81]	0.87
[55; 59]			3.30	[0.35; 31.39]	0.30
[60; 64]			0.79	[0.09; 6.93]	0.83
[65; 69]			1.10	[0.08; 14.74]	0.95
**Practice location**	1.55	> 0.20	-	-	-
**Practice structure**	2.16	> 0.20	-	-	-
**Number of patients per week registered at the GP practice**	0.97	> 0.20	-	-	-
**Number of RA patients per year**	2.89	> 0.20	-	-	-
**Self-assessment of knowledge about the RA therapy strategy**	1.09^a^	> 0.20	-	-	-
**Knowledge of the new guidelines for RA treatment**	0.02	> 0.20	-	-	-
**Evaluation of medication adherence at each visit**	2.12	0.15	1.37	[0.66; 2.84]	0.40
**Causes of medication non-adherence already identified**	6.05	0.01	1.95	[1.03; 3.69]	0.04*
**Patient management sufficiently coordinated between healthcare professionals in RA**	2.66	0.10	0.76	[0.40; 1.45]	0.76
**Sufficient transmissions by the rheumatologist regarding follow-up, adjustments, and monitoring of the treatment**	0.34	> 0.20	-	-	-
**Interest in training in the practice of motivational interviewing to improve medication adherence**	32.07	< 0.01	3.83	[1.91; 7.69]	< 0.01*
**Interest in providing additional time to explore and improve medication adherence with financial compensation**	32.55	< 0.01	3.11	[1.58; 6.13]	< 0.01*

^*^a *P* value < 0.05 was considered as significant

Abbreviations: *GP* general practitioner, *RA* rheumatoid arthritis, *OR* odd ratio, *CI* confidence interval

## Discussion

### Practice and knowledge of GPs

GPs reported they played an important role in the care of RA patients for disease detection and monitoring, and management of comorbidities. Nevertheless, most of them considered their role in the therapeutic adjustment (dosage of corticosteroids or DMARDs according to tolerance and effectiveness) to be limited. In a qualitative study conducted in 2015, GPs have been reported to consider intensive treatment initiation to be the expertise of rheumatologists, nevertheless the lack of GP involvement has been identified as a key barrier to intensive treatment [11].

In the context of RA, a poor medication adherence has been frequently reported in the literature [12, 13]. This issue seemed to be taken into account by the GPs participating to the present survey as almost three quarters of them reported evaluating patient adherence at each visit. Many of them also confirmed that they already identified the causes of non-adherence, which were mostly related to the adverse effects suffered and beliefs about drugs. These factors are consistent with previous reports [14, 15].

### Perception of patient knowledge

GPs estimated herein patients had a low level of knowledge regarding their treatment, particularly on the action mechanism, adverse effects, vaccinations, and biosimilars, which has been proposed to complicate the shared decision-making process regarding RA management [16]. Our results reinforced the important role of GPs in primary care in the follow-up of RA patients. In a precedent study, GPs have reported their wish to provide disease and treatment education in order to motivate patients to continue medication intake [11].

### Obstacles encountered and need for training

The latest French SFR recommendations have been published in 2018 [4] and more than 9 in 10 GPs admitted not being aware of them. Despite the important role of GPs in patient management highlighted in these guidelines, this can be explained by the multitude of diseases managed by GPs. Management guidelines for rheumatic diseases are published more often in specialty rheumatology literature than in general medical journals [17]. The limited period between the publication of the guidelines and the survey could also explain the lack of awareness of the GPs interviewed. The main difficulties mentioned by GPs were related to targeted therapies (synthetics and biologicals). Contrary to the recommendations, the majority of GPs considered that targeted therapies should be used preferentially in monotherapy. They felt uncomfortable with adverse effects, vaccinations, and biosimilars, even though they considered patient knowledge regarding these topics was insufficient. Among these topics, the importance of vaccination has been particularly recognised in previous reports [18, 19].

Given the difficulties experienced, it would seem interesting to propose a training course for GPs. Keyszer et al. have considered a high level of competence among GPs as essential in the primary care management of patients with rheumatic diseases [20]. Despite the frequency of rheumatic diseases in primary care, the formal training of GPs in rheumatology remains limited [17]. An adequate formation would be a condition for the involvement of GPs in a multidisciplinary programme to improve patient care in RA patients. Knowledge and compliance with the recommendations (for instance, early diagnosis, management of comorbidities, identification of non-adherence factors, and drug monitoring…) is an issue for improving patient care and coordination between primary care and rheumatology.

### Multidisciplinary programme

Herein, more than half of the GPs estimated the coordination between health professionals in the context of RA was insufficient. These barriers have already been described by GPs who considered the cooperation with rheumatologists to be inadequate [21] and the shared-care arrangements to be limited [11]. They felt they lost their patient when referring to a rheumatologist [11]. Suter *et al.* have showed that only 55–80% of all specialists communicated back to GPs [22]. In our study, a quarter of the GPs had already encountered difficulties in obtaining specialised rheumatology opinions. A common language between rheumatologists and GPs is often perceived as absent by some authors [21]. Meyfroidt et al*.* have also estimated that the lack of communication and collaboration with rheumatologists could be bettered by the use of a structured electronic platform [11]. A regular communication has been identified as an important facilitator of interprofessional collaboration [23].The benefits of a secure e-mail system for sharing information with other professionals were widely reported in the present study. Optimal treatment requires a multidisciplinary approach with an intensive programme of re-evaluation and treatment adjustments, which is impossible without a strong cooperation between rheumatologists and GPs [21]. This type of cooperation has been shown to be beneficial in reducing the number of referrals to rheumatologists and the waiting times for non-urgent visits [24].

In our survey, the majority of GPs were willing to participate in a multidisciplinary programme in collaboration with hospital professionals and community pharmacists to improve patient adherence. The factors related to the GP motivation were causes of non-adherence already identified, interest in training in the practice of motivational interviewing, and interest in providing additional time to explore and improve medication adherence with financial compensation. In other diseases, models of collaboration in primary care are being developed [25]. Several studies have showed the recognition of pharmacist and GP roles in collaborative care approaches [26, 27]. Once the barriers between pharmacists and GPs were addressed, collaborations were linked to improved patient outcomes [28, 29]. In the context of an increasing burden on primary healthcare services, effective multidisciplinary collaboration is crucial for sustaining high quality care [23]. Nevertheless, interaction with specialists for therapeutic optimisation should not be neglected in order to obtain a transparent continuity of care [27].

For discussion purposes, this study was conducted before the covid-19 pandemic which may have positively or negatively affected GPs' willingness and interest in being involved in this type of multidisciplinary programme.

### Strength and limitations

The study findings provided a picture of the current knowledge and practice of GPs in RA. The results highlighted some of the factors related to GPs' motivation to get involved in a multidisciplinary programme in RA. Several limitations need to be acknowledged, among which the low response rate and selection bias inherent to survey studies. However, this response rate was similar to those found in other survey studies including French GPs [30, 31]. This low rate of participation can be explained, for instance, by the reported difficulty to involve GPs in research [32]. In order to motivate GPs to participate in studies, a diversification in the types of studies, domains, and themes seems to be important [33]. The topic of RA in primary care remains a question of interest worldwide. Promoting multidisciplinary collaborations and assessing medication adherence is recognised in the guidelines [4, 34]. Similarly, the lack of knowledge and the inadequate collaboration with rheumatologists identified herein by GPs have also been acknowledged by other authors in different countries [11, 35, 36]. In order to encourage GPs to respond to our survey, a leaflet on the main characteristics of DMARDs was provided at the end of the questionnaire. A reminder was also sent to all potential participants during the questionnaire distribution period to optimise participation. Secondly, the low response rate might be related to the length of the questionnaire. Indeed, 136 GPs started the questionnaire without completing it. Finally, the collection of information concerning the GPs’ postgraduate experience or training in rheumatology may have been interesting to document the interest of GPs and explain a selection bias.

## Conclusions

Altogether, our results suggest that GPs have already a role in the management of RA patients. They are motivated to become more involved, especially in a multidisciplinary programme. Nevertheless, they need professional education about RA treatment and training in motivational interviews before getting involved in an intervention to improve patient care. A qualitative study of GPs' and rheumatologists’ perceptions is required in order to analyse more precisely the barriers and facilitators that influence the implementation of a multidisciplinary programme, and to clarify their respective roles.

## Supplementary Information

**Additional file 1. **Cover letter and questionnaire (English-translated version).

**Additional file 2. **Cover letter and questionnaire (original French version).

**Additional file 3. **CHERRIES Checklist for Online Questionnaires.

## Data Availability

Data that support the findings in the current study are available from the corresponding author (anne-laure.yailian@chu-lyon.fr) on reasonable request.
