# Supplementary material for: Practices among General Practitioners in Rheumatoid Arthritis (GEPRA-I): results of a region-wide online survey
Source: BMC Prim Care. 2022 Jun 3;23:144. doi: 10.1186/s12875-022-01744-5 (PMC9164358; doi:10.1186/s12875-022-01744-5)
Supplement: Supplementary file 3 — Additional file 3. CHERRIES Checklist for Online Questionnaires. [file 12875_2022_1744_MOESM3_ESM.docx]

**Additional file 3: CHERRIES Checklist for Online Questionnaires**

| **Checklist item** | **Details** |
| --- | --- |
| Study Design: Describe target population & sample frame. Is the sample a convenience sample? | All GPs in the Auvergne-Rhône-Alpes region registered by the URPS were invited to participate.  This was not a convenience sample.  (page 6) |
| IRB Approval: Has the study been approved by an IRB? | The study was approved by the General Medicine Ethics Committee at the *Université Claude Bernard Lyon 1*.  (page 5) |
| Informed consent:  Describe the informed consent process. Where were the participants told the length of time of the survey, which data were stored & where/for how long, who the investigator was, purpose of the study? | The introductory paragraph at the start of the survey:   - Described the purpose of our study - Informed participants that no identifying data was collected - Provided the investigator’s contact info   They were also informed that participating in the survey implied their consent for their answers to be used for research.  (additional file 1) |
| Data protection: If any personal information was collected or stored, describe what mechanisms were used to protect unauthorized access. | No personal information was collected or stored.  The study was approved by the data protection officer of the *Université Claude Bernard* *Lyon* *1*.  (page 6) |
| Development and testing | The survey was reviewed for content validity by 4 rheumatologists. It was pilot tested by a group of 4 GPs.  No major adjustment was necessary.  (pages 5 and 6) |
| Open survey versus closed survey | This was not an open survey.  Participants had to enter their password to access the survey sent by email.  (page 6) |
| Contact mode: Indicate whether or not the initial contact with the potential participants was made on the Internet. | Contact was made via email.  (page 6) |
| Advertising the survey: How/where was the survey announced or advertised? It is important to know the wording of the announcement as it will heavily influence who chooses to participate. | The survey was advertised via email sent by the URPS physicians of the AuRA region.  ‘We would like to invite you to participate in a survey on practices and knowledge related to the therapeutic follow-up of your RA patients. The aim of our survey is to analyse your possible difficulties in the follow-up in order to propose improvements. The information derived from this study will additionally help us to propose a multidisciplinary program in RA.’  (page 6) |
| Web/Email:  State the type of e-survey (e.g. one posted on a Web site or one sent out through e-mail). If it is an e-mail survey, were the responses entered manually into a database, or was there an automatic method for capturing responses? | The survey was sent out through email. The e-survey was hosted on Claroline Connect.  One reminder was sent to increase the response rate.  In Claroline Connect, responses were automatically captured into a database.  (pages 5 and 6) |
| Context:  Describe the Web site (for mailing list/newsgroup) in which the survey was posted. | N/A.  Participants could access the site via the link sent to their email.  (page 6) |
| Mandatory/voluntary: Was it a mandatory survey to befilled in by every visitor who wanted to enter the Web site, or was it a voluntary survey? | It was a voluntary survey.  (page 6) |
| Incentives:  Were any incentives offered  (e.g., monetary, prizes, or non-  monetary incentives such as an  offer to provide the survey results)? | No financial incentive was offered.  Participants were offered access to a summary form on disease-modifying therapies for RA and the latest recommendations of the French Society of Rheumatology regarding the management of this disease.  (page 6) |
| Time/Date:  In what timeframe were the data collected? | The data were collected over a period of 3 months.  (page 6) |
| Randomisation of items:  To prevent biases items can be  randomized or alternated | The items were not randomized as this may have confused the participants.  (page 6) |
| Adaptive questioning:  Use adaptive questioning (certain items, or only conditionally displayed based on responses to other items) to reduce number and complexity of the questions. | There were 2 adaptive questions based on the response to the previous question.  (page 6) |
| Number of Items:  What was the number of questionnaire items per page? The number of items is an important factor for the completion rate | Number of questions per section:  Section 1: 8 questions (1 page)  Section 2: 20 questions (2 pages)  Section 3: 7 questions (1 page)  (additional file 1) |
| Number of Screens:  Over how many pages was the questionnaire distributed? The number of items is an important factor for the completion rate | Total of 6 pages  1 page of introduction  4 pages for survey questions  1 page for potential comments  (additional file 1) |
| Completeness check: Were consistency or completeness checks before questionnaire submission? An alternative is to check for completeness after the questionnaire has been submitted (and highlight mandatory items). | There was a completeness check at the end of each page.  All answers were mandatory, except 2 open-ended questions.  (page 6) |
| Unique site visitor | Unique site visitor was defined by the absence of duplicate socio-demographic data.  (page 7) |
| View rate (Ratio of unique survey visitors/unique site visitors) | N/A |
| Participation rate | Participation rate was defined as the ratio of [the number of GPs who had started the questionnaire] / [the number of GPs who received the email]  (page 7) |
| Completion rate (Ratio of users who finished the survey/users who agreed to participate): This is only relevant if there is a separate “informed consent” page or if the survey goes over several pages. This is a measure for attrition. | Completion rate was defined as the ratio of [the number of GPs who completed the survey] / [the number of GPs who started the questionnaire].  (page 7) |
| Cookies used:  Indicate whether cookies were used to assign a unique user identifier to each client computer. | We did not incorporate these elements into our survey. |
| IP check:  Indicate whether the IP address of the client computer was used to identify potential duplicate entries from the same user. | We did not incorporate these elements into our survey. |
| Log file analysis:  Indicate whether other techniques to analyze the log file for identification of multiple entries were used. If so, please describe. | We decided not to require log-in as this may have reduced the participation rate. |
| Registration:  In “closed” (non-open) surveys, users need to login first and it is easier to prevent duplicate entries from the same user. | N/A |
| Handling of incomplete questionnaires:  Were only completed questionnaires analysed? | Only completed questionnaires were analysed.  (page 7) |
| Statistical correction:  Indicate whether any methods such as weighting of items or propensity scores have been used to adjust for the non-representative sample; if so, please describe the methods. | No statistical correction was used to adjust for the non-representative sample. |
