# Supplementary material for: Practices among General Practitioners in Rheumatoid Arthritis (GEPRA-I): results of a region-wide online survey
Source: BMC Prim Care. 2022 Jun 3;23:144. doi: 10.1186/s12875-022-01744-5 (PMC9164358; doi:10.1186/s12875-022-01744-5)
Supplement: Supplementary file 1 — Additional file 1. Cover letter and questionnaire (English-translated version). [file 12875_2022_1744_MOESM1_ESM.docx]

**Additional File 1: Cover letter and questionnaire (English-translated version)**

Dear Mrs/Mr,

I am currently working on the optimisation of the therapeutic management of patients with rheumatoid arthritis (RA) in primary care and hospital.

This pathology is known to be the most frequent chronic inflammatory rheumatism in adults. It used to be very disabling, whereas today it can be controlled as an increasing number of effective therapies (for instance, biotherapies, biosimilars, and new targeted therapies *per os*) are available. Nevertheless, a lack of therapeutic adherence is recognised in RA, as in other chronic diseases, and reduces the expected benefits. This leads to therapeutic failures, a progression of the disease activity, and, therefore, a deterioration of patient health status.

I therefore propose a questionnaire to analyse your practices and knowledge related to the therapeutic follow-up of the RA patients under your care. This study lies in the context of a willingness to strengthen the relationship between primary care and hospital, and of updated recommendations in the disease and the availability of new molecules. The main objective is to analyse your possible difficulties in the follow-up in order to propose improvements.

The questionnaire is addressed to all the general practitioners in the Auvergne-Rhône-Alpes region. It is anonymous and your answers are strictly confidential. Your answers are expected for the questions indicated as mandatory.

It takes about 10 minutes to complete and the results of the survey will be sent to you afterwards.

Your participation in this survey implies your consent to the use of your answers for research purposes.

At the end of this questionnaire, you will be offered access to a summary form on disease-modifying therapies for RA as well as the latest recommendations of the French Society of Rheumatology regarding the management of this disease. I hope that these documents will provide you with some useful information for your practice.

You are welcome to contact the investigators of this research at [GEPRA.AURA@gmail.com](mailto:GEPRA.AURA@gmail.com) if you have any questions regarding this survey.

Thank you very much for your contribution to this work.

*mandatory items

**SECTION 1 (1 page; 8 questions): Demographic and practice characteristics**

1. What is your gender?*
2. What is your age?*

- 25-29 years
- 30-34 years
- 35-39 years
- 40-44 years
- 45-49 years
- 50-54 years
- 55-59 years
- 60-64 years
- 65-69 years
- 70-74 years

3. What is your practice department?*

- Ain
- Allier
- Ardèche
- Cantal
- Drome
- Isère
- Loire
- Haute Loire
- Puy de Dôme
- Rhône
- Savoie
- Haute Savoie

4. What is your practice location?*

- Rural area
- Semi-rural area
- Urban area

5. What is your practice structure?*

- Medical practice alone
- Medical group practice
- Multidisciplinary health centre
- Employee in a health care institution
- Other

6. How long have you been established?*

- Less than 5 years
- Between 5 and 10 years
- Between 10 and 20 years
- Between 20 and 30 years
- Over 30 years

7. In what year did you complete your thesis?*

- Before 1985
- Between 1985 and 1990
- Between 1991 and 1995
- Between 1996 and 2000
- Between 2001 and 2005
- Between 2006 and 2010
- Between 2011 and 2015
- After 2015

8. How many patients do you see per week?*

- Less than 50 patients
- Between 50 and 100 patients
- Between 100 and 200 patients
- Over 200 patients

**SECTION 2 (2 pages; 20 questions): practice and knowledge in RA**

9. How many patients with rheumatoid arthritis (RA) have you received in the past year?*

- No patient
- Less than 5 patients
- Between 5 and 15 patients
- Over 15 patients

10. How often do you receive these patients on average?*

- More than every 3 months
- Every 3 to 6 months
- Every 6 to 12 months
- Less than every 12 months

11. Where do you think specialist rheumatology follow-up for your RA patients is carried out?*

- Mostly in hospitals
- Mostly in primary care
- Both

12. Have you ever had difficulties in getting expert opinion about RA?*

- Yes
- No

13. How important is your role in the management of patients with RA?*

|  | Not at all important | Not very important | Relatively important | Very important | Extremely important |
| --- | --- | --- | --- | --- | --- |
| In diagnosing the disease |  |  |  |  |  |
| In monitoring the disease |  |  |  |  |  |
| In monitoring the effectiveness treatment |  |  |  |  |  |
| In monitoring the tolerance treatment |  |  |  |  |  |
| In renewing DMARDs |  |  |  |  |  |
| In adjusting treatments (corticosteroids or DMARDs) |  |  |  |  |  |
| In managing co-morbidities |  |  |  |  |  |
| In supporting patients in their treatment management |  |  |  |  |  |

14. Do you assess the medication adherence of these patients during the consultation?*

- Yes
- No

15. Have you ever identified any causes of non-adherence in these patients?*

- Yes
- No

16. If yes to question 15, what were the causes of non-adherence (the 2 main ones, briefly in a few words)?

__________________________________________________________________________________________________________________________________________

17. Do you believe that connected tools can improve tolerance and adherence?*

- Yes
- No

18. In which aspects do you experience difficulties in the management of patients with RA? Multiple answers possible*

- I do not face any particular difficulty
- Practical management of medicines (e.g. storage)
- Benefits of certain diets (e.g. gluten-free, lactose-free)
- Instructions for particular situations (e.g. infections)
- Potential adverse effects
- Information on biosimilars
- Contraindicated vaccines
- Recommended vaccines
- Treatment strategies (choice of medicines, monotherapy or not)

19. Do you regularly discuss health and diet recommendations with RA patients (including physical activity, smoking cessation, healthy diet, etc.)?*

- Yes
- No

20. Do you discuss/investigate the following items? (tick as appropriate) - Multiple answers possible*

- Cardiovascular risk factors
- Vaccination status
- Cancer screening tests
- Oral health
- Risk of osteoporosis
- Pulmonary damage
- Digestive disorder
- Depression
- None of the items above

21. Regarding DMARDs, what is your patients' level of knowledge about …?*

|  | Very Insufficient | Insufficient | No opinion | Sufficient | Excellent |
| --- | --- | --- | --- | --- | --- |
| Mechanism of action |  |  |  |  |  |
| Potential adverse effects and associated behaviours |  |  |  |  |  |
| Frequency of administration |  |  |  |  |  |
| Vaccination status |  |  |  |  |  |
| Biosimilars |  |  |  |  |  |
| Self-injection |  |  |  |  |  |

22. On a scale of 0 to 10, how would you rate your knowledge of the current treatment strategy for RA?*

0	1	2	3	4	5	6	7	8	9	10

23. For the new synthetic targeted therapies XELJANZ® (tofacitinib) and OLUMIANT® (baracitinib), check the item(s) you are most comfortable with:*

- Their mechanism of action
- Their place in the therapeutic strategy
- Their administration regimen
- Their safety profile
- Precautions for use and contraindications
- Their possible drug interactions
- Their associated risks and procedures to follow
- I am not comfortable with the above items

24. For injectable targeted therapies (=biotherapies), tick the item(s) you are most comfortable with:*

- Their mechanism of action
- Their place in the therapeutic strategy
- Their administration regimen
- Their safety profile
- Precautions for use and contraindications
- Their possible drug interactions
- Their associated risks and procedures to follow
- Their biosimilars
- I am not comfortable with the above items

25. Were you aware of the 2018 update in the recommendations for the management of RA?*

- Yes
- No

26. In your opinion, targeted therapies (biological or synthetic) should be used preferentially:*

- In monotherapy
- In association with methotrexate

27. Have you ever experienced adverse reactions in a patient receiving targeted therapy (biotherapy, or synthetic treatments such as XELJANZ® or OLUMIANT®)?*

- Yes
- No

28. If yes to question 27, which ones (the 2 most frequent, briefly in a few words)?

__________________________________________________________________________________________________________________________________________

**SECTION 3 (1 page; 7 questions): Community-hospital and multidisciplinary collaboration**

29. Do you think that the follow-up of patients with RA is sufficiently coordinated between health professionals?*

- Yes
- No

30. Do you receive sufficient feedback from the specialist regarding follow-up, changes, and monitoring of background treatment?*

- Yes
- No

31. Would you be willing to participate in a multi-professional programme with hospital professionals and community pharmacists to improve patient adherence?*

- Yes
- No

32. If no to question 31, why not? Multiple answers possible*

- Lack of time
- Lack of motivation
- Lack of training
- Lack of willingness of patients
- Too few patients involved in my practice
- Lack of added value
- Other reason(s)

33. Do you find it useful to have a secure email system to communicate with other professionals about your patients?*

- Yes
- No

34. Would you like to be trained in motivational interviewing for adherence?*

- Yes
- No

35. During a consultation with one of your patients, would you be willing to spend additional time exploring and reinforcing adherence, with appropriate training and remuneration?*

- Yes
- No

**Thank you for completing the survey.**

For any question or comment, please send an email to GEPRA.AURA@gmail.com
