# Supplementary material for: Practices among General Practitioners in Rheumatoid Arthritis (GEPRA-I): results of a region-wide online survey
Source: BMC Prim Care. 2022 Jun 3;23:144. doi: 10.1186/s12875-022-01744-5 (PMC9164358; doi:10.1186/s12875-022-01744-5)
Supplement: Supplementary file 2 — Additional file 2. Cover letter and questionnaire (original French version). [file 12875_2022_1744_MOESM2_ESM.docx]

**Additional File 2: Cover letter and questionnaire (original French version)**

Madame, Monsieur,

Je travaille actuellement sur l'optimisation de la prise en charge thérapeutique des patients atteints de polyarthrite rhumatoïde (PR) en ville et à l'hôpital.

Cette pathologie est reconnue pour être le rhumatisme inflammatoire chronique le plus fréquent chez l’adulte. Elle était autrefois très invalidante alors qu’aujourd’hui elle peut être contrôlée avec des thérapeutiques efficaces, de plus en plus nombreuses (biothérapies, biosimilaires, nouvelles thérapies ciblées per os…). Néanmoins, un défaut d’adhésion thérapeutique est reconnu dans la PR, à l’instar d’autres maladies chroniques, et diminue les bénéfices escomptés. Cela entraîne des échecs thérapeutiques, une progression de l’activité de la maladie et donc une détérioration de l’état de santé du patient.

Je vous propose ainsi un questionnaire pour analyser vos pratiques et connaissances en lien avec le suivi thérapeutique de vos patients atteints de PR. Cette étude s’inscrit dans une volonté de renforcement du lien ville-hôpital dans un contexte d'actualisation des recommandations dans la pathologie et de mise à disposition de nouvelles molécules. L’objectif principal est d’analyser vos éventuelles difficultés dans le suivi afin de proposer des axes d’amélioration.

Le questionnaire s'adresse à tous les médecins généralistes de la région Auvergne-Rhône-Alpes. Il est anonyme et vos réponses sont strictement confidentielles. Vos réponses sont attendues pour les questions indiquées comme obligatoires.

Le temps de remplissage est estimé à 10 minutes et les résultats de l’enquête vous seront communiqués par la suite.

Votre participation à cette enquête implique votre consentement à l'utilisation de vos réponses à des fins de recherche.

A la fin de ce questionnaire, il vous sera proposé l’accès à une fiche-mémo concernant les traitements de fond dans la PR ainsi que les dernières recommandations de la Société Française de Rhumatologie concernant la prise en charge de cette pathologie. J’espère que ces documents pourront vous apporter quelques informations utiles à vos pratiques.

Vous pouvez contacter les investigateurs de cette recherche à l'adresse GEPRA.AURA@gmail.com si vous avez des questions concernant cette enquête.

En vous remerciant chaleureusement pour votre contribution à ce travail.

*éléments obligatoires

**PARTIE 1 (1 page ; 8 questions): Caractéristiques démographiques et pratiques des médecins**

1. Quel est votre sexe ?*
2. Quel est votre âge ?*

- 25-29 ans
- 30-34 ans
- 35-39 ans
- 40-44 ans
- 45-49 ans
- 50-54 ans
- 55-59 ans
- 60-64 ans
- 65-69 ans
- 70-74 ans

3. Quel est le département de votre lieu d'exercice ?*

- Ain
- Allier
- Ardèche
- Cantal
- Drome
- Isère
- Loire
- Haute Loire
- Puy de Dôme
- Rhône
- Savoie
- Haute Savoie

4. Quelle est votre zone d’exercice ?*

- Zone rurale
- Zone semi-rurale
- Zone urbaine

5. Quel est votre mode d’exercice ?*

- Cabinet seul
- Cabinet de groupe
- Maison multidisciplinaire
- Salarié dans un établissement de santé
- Autre

6. Depuis combien de temps êtes-vous installé ?*

- Moins de 5 ans
- Entre 5 et 10 ans
- Entre 10 et 20 ans
- Entre 20 et 30 ans
- Plus de 30 ans

7. En quelle année avez-vous passé votre thèse d'exercice ?*

- Avant 1985
- Entre 1985 et 1990
- Entre 1991 et 1995
- Entre 1996 et 2000
- Entre 2001 et 2005
- Entre 2006 et 2010
- Entre 2011 et 2015
- Après 2015

8. Combien de patients voyez-vous par semaine ?*

- Moins de 50 patients
- Entre 50 et 100 patients
- Entre 100 et 200 patients
- Plus de 200 patients

**PARTIE 2 (2 pages ; 20 questions): Pratiques et connaissances dans la polyarthrite rhumatoïde**

9. Combien de patients atteints de PR avez-vous vu en consultation pendant l'année écoulée ?*

- Aucun
- Moins de 5 patients
- Entre 5 et 15 patients
- Plus de 15 patients

10. A quelle fréquence voyez-vous ces patients en moyenne average?*

- Plus souvent que tous les 3 mois
- Tous les 3 à 6 mois
- Tous les 6 à 12 mois
- Moins d’une fois par an

11. Selon vous, où est effectué le suivi spécialisé de rhumatologie pour vos patients atteints d'une PR ??*

- Majoritairement à l’hôpital
- Majoritairement en ville
- Les deux

12. Avez-vous déjà rencontré des difficultés pour obtenir un avis spécialisé à propos de la PR?*

- Oui
- Non

13. Quelle est l'importance de votre rôle dans la prise en charge des patients atteints de PR?*

|  | Pas du tout important | Peu important | Relativement important | Très important | Extrêmement important |
| --- | --- | --- | --- | --- | --- |
| Dans la détection de la maladie |  |  |  |  |  |
| Dans le suivi de la maladie |  |  |  |  |  |
| Dans le suivi d’efficacité du traitement |  |  |  |  |  |
| Dans le suivi de tolérance du traitement |  |  |  |  |  |
| Dans le renouvellement des traitements de fond |  |  |  |  |  |
| Dans les ajustements thérapeutiques (corticoïdes ou traitements de fond)) |  |  |  |  |  |
| Dans la prise en charge des comorbidités |  |  |  |  |  |
| Dans l'aide du patient pour la gestion de son traitement |  |  |  |  |  |

14. Evaluez-vous l'adhésion thérapeutique de ces patients lors de la consultation?*

- Oui
- Non

15. Avez-vous déjà identifié des causes de non-adhésion médicamenteuse chez ces patients?*

- Oui
- Non

16. Si oui à la question 15, quelles étaient les causes de non-adhésion (les 2 principales, brièvement en quelques mots) ?

__________________________________________________________________________________________________________________________________________

17. Pensez-vous que les outils connectés peuvent permettre d'améliorer la tolérance et l'adhésion thérapeutique?*

- Oui
- Non

18. Sur quels aspects rencontrez-vous des difficultés dans la prise en charge des patients atteints de PR? Plusieurs réponses possibles*

- Je ne rencontre pas de difficulté particulière
- Gestion pratique des médicaments (ex : conservation
- Utilité de certains régimes (ex : sans gluten, sans lactose)
- Conduite à tenir si situation particulière (ex : infections)
- Effets indésirables éventuels
- Information sur les biosimilaires
- Vaccins contre-indiqués
- Vaccins recommandés
- Stratégies thérapeutiques (choix des molécules, monothérapie ou non)

19. Discutez-vous régulièrement de conseils hygiéno-diététiques avec les patients atteints de PR (dont activité physique, arrêt du tabac, alimentation équilibrée...)?*

- Oui
- Non

20. Abordez-vous / Recherchez-vous ? (cocher le cas échéant) – plusieurs réponses possibles*

- Les facteurs de risque cardiovasculaire
- Le statut vaccinal
- La mise à jour des dépistages néoplasiques
- L’état buccodentaire
- Le risque d’ostéoporose
- Une atteinte pulmonaire
- Une atteinte digestive
- Une dépression
- Aucun des items ci-dessus

21. Concernant les traitements de fond, quel est le niveau de connaissance de vos patients sur …?*

|  | Très insuffisant | Insuffisant | Sans avis | Satisfaisant | Excellent |
| --- | --- | --- | --- | --- | --- |
| Le mécanisme d’action |  |  |  |  |  |
| **les éventuels effets indésirables et conduites à tenir associées** |  |  |  |  |  |
| Le rythme d’administration |  |  |  |  |  |
| Le statut vaccinal |  |  |  |  |  |
| Les biosimilaires |  |  |  |  |  |
| L’auto-injection |  |  |  |  |  |

22. Sur une échelle de 0 à 10, comment évaluez-vous vos connaissances concernant la stratégie thérapeutique actuelle de la PR?*

0	1	2	3	4	5	6	7	8	9	10

23. Concernant les nouvelles thérapies ciblées synthétiques XELJANZ® (tofacitinib) et OLUMIANT® (baracitinib), cochez le ou les item(s) pour lesquels vous êtes le plus à l'aise :*

- Leur mécanisme d’action
- Leur place dans la stratégie thérapeutique
- Leur schéma d’administration
- Leur profil de tolérance
- Leurs précautions d’emploi et contre-indications
- Leurs interactions médicamenteuses éventuelles
- Leurs risques associés et conduites à tenir
- Je ne me sens pas à l’aise avec les items ci-dessus

24. Concernant les thérapies ciblées injectables (=biothérapies), cochez le ou les item(s) pour lesquels vous êtes le plus à l'aise :*

- Leur mécanisme d’action
- Leur place dans la stratégie thérapeutique
- Leur schéma d’administration
- Leur profil de tolérance
- Leurs précautions d’emploi et contre-indications
- Leurs interactions médicamenteuses éventuelles
- Leurs risques associés et conduites à tenir
- Leurs biosimilaires
- Je ne me sens pas à l’aise avec les items ci-dessus

25. Aviez-vous connaissance de l'actualisation des recommandations de prise en charge de la PR en 2018 ?*

- Oui
- Non

26. Selon vous, les thérapeutiques ciblées (biologiques ou synthétiques) doivent être utilisées préférentiellement:*

- In monotherapy
- In association with methotrexate

27. Avez-vous déjà rencontré des effets indésirables chez un patient sous thérapie ciblée (biothérapie, ou traitements synthétiques type XELJANZ® ou OLUMIANT®) ??*

- Oui
- Non

28. Si oui à la question 27, lesquels (les 2 plus fréquents, brièvement en quelques mots)?

__________________________________________________________________________________________________________________________________________

**PARTIE 3 (1 page ; 7 questions): Lien ville-hôpital et collaboration pluriprofessionnelle**

29. Pensez-vous que le suivi des patients atteints de PR est suffisamment coordonné entre professionnels de santé?*

- Oui
- Non

30. Recevez-vous suffisamment de transmissions par le spécialiste concernant le suivi, les modifications et la surveillance du traitement de fond?*

- Oui
- Non

31. Seriez-vous prêt(e) à participer à un programme pluriprofessionnel en lien avec les professionnels de l'hôpital et le pharmacien de ville pour améliorer l'adhésion thérapeutique de vos patients?*

- Oui
- Non

32. Si non à la question 31, pourquoi? Plusieurs réponses possibles*

- Manque de temps
- Manque de motivation
- Manque de formation
- Manque de volonté des patients
- Peu de patients concernés dans ma patientèle
- Manque de plus-value
- Autre(s) raison(s)

33. Trouvez-vous utile de disposer d'une messagerie électronique sécurisée pour vos échanges avec d'autres professionnels à propos de vos patients?*

- Oui
- Non

34. Aimeriez-vous recevoir une formation pour la pratique d'entretiens motivationnels pour le renforcement de l'adhésion thérapeutique?*

- Oui
- Non

35. Lors d'une consultation avec un de vos patients, seriez-vous prêt(e) à consacrer un temps additionnel pour explorer et renforcer l'adhésion thérapeutique, avec une formation et une rémunération adaptées?*

- Oui
- Non

**Merci d'avoir répondu à l'enquête.**

Pour toute question ou commentaire, veuillez envoyer un courriel à GEPRA.AURA@gmail.com.
